# Comparison of the reconstruction of through-and-through cheek defects involving the labial commissure following tumor resection using four types of local and pedicle flaps

**DOI:** 10.1186/s13005-019-0196-6

**Published:** 2019-05-16

**Authors:** Wei-liang Chen, Yan Wang, Bin Zhou, Juan-kun Liao, Rui Chen

**Affiliations:** Department of Oral and Maxillofacial Surgery, Sun Yat-sen Memorial Hospital, Sun Yat-sen University, 107 Yan-jiang Road, Guangzhou, 510120 China

**Keywords:** Buccal squamous cell carcinoma, Cheek defect, Pedicle flaps, Abbe–Estlander flap, Supraclavicular flap, Pectoralis major muscle flap, Trapezius myocutaneous flap

## Abstract

**Background:**

The reconstruction of through-and-through cheek defects involving the labial commissure following cancer ablation is a surgical challenge.

**Methods:**

This study evaluated 35 patients with buccal squamous cell carcinoma (SCC) involving the labial commissure who underwent Abbe–Estlander (A-EF), folded extended supraclavicular fasciocutaneous island (SFIF), folded pectoralis major muscle (PMMF), or folded extended vertical lower trapezius island myocutaneous (TIMF) flap reconstruction of through-and-through cheek defects involving the labial commissure following radical resection.

**Results:**

The A-EF and SFIF groups differed significantly (*P* < 0.05) from the PMMF and TIMF groups in terms of tumor clinical stage and type of treatment. The inner PMMF (median 6.3 × 4.5) and TIMF (median 9.8 × 6.7) skin paddle dimensions were larger than those of the A-EF (median 1.8 × 2.2) and SFIF (median 5.5 × 4.3) groups (*P* < 0.05). The outer PMMF (median 6.3 × 6.6) and TIMF (median 9.8 × 13.2) dimensions were larger than those of the A-EF (median 1.8 × 3.8) and SFIF (median 5.5 × 4.6) groups (*P* < 0.05). The esthetic results, orbicularis oris function, and speech function were significantly (*P* < 0.05) better in the A-EF group than in the SFIF, PMMF, and TIMF groups. The patients were followed for 6–38 months (median 26.8, 25.0, 22.1, and 20.8 months in the A-EF, SFIF, PMMF, and TIMF groups, respectively). At the final follow-up, 4 (80.0%) patients in the A-EF, 7 (87.5%) in the SFIF, 5 (55.6%) in the PMMF, and 5 (38.4%) in the TIMF groups were alive with no disease; 1 (20.0%), 1 (22.2%), 2 (22.2%), and 4 (30.8%) patients, respectively, were alive with disease; and 2 (22.2%) patients in the PMMF and 4 (30.8%) in the TIMF group had died of local recurrence or distant metastases at between 9 and 38 months. There was a significant survival difference in the A-EF and SFIF groups compared with the PMMF and TIMF groups (*P* < 0.05).

**Conclusions:**

The A-EF is suitable for reconstructing defects of clinical stage II disease; the SFIF for clinical stage II or III disease; the PMMF for clinical stage III or IV; and the TIMF for clinical stage rCS III or rCS IV disease.

## Introduction

Reconstruction of through-and-through cheek defects involving the labial commissure following cancer ablation is a surgical challenge. Several methods for reconstructing small- to medium-sized defects cheek and labial commissure defects have been reported, such as the Karapandzic [1], Estlander [2], Abbe–Estlander flap (A-EF) [3], double full-thickness cheek rhomboidal [4], and facial artery musculomucosal flaps [5]. For the reconstruction of large through-and-through cheek defects involving the oral commissure, several free flaps have been reported, including chimeric flaps from the thigh lateral femoral circumflex system [6], the radial forearm free flap [7], and double-paddle peroneal chimeric flap [8]. Recently, we developed the folded extended supraclavicular fasciocutaneous island flap (SFIF) based on the transverse cervical vessels for reconstructing through-and-through cheek defects [9]. The folded pectoralis major muscle flap (PMMF) based on the thoracoacromial vessels [10], and folded extended vertical lower trapezius island myocutaneous flap (TIMF) based on the transverse cervical vessels can also be used [11, 12]. This study compares the outcomes of A-EF, SFIF, PMMF, and TIMF pedicle flaps for reconstructing through-and-through cheek defects involving the labial commissure following cheek cancer ablation.

## Patients and methods

The study evaluated 35 patients with buccal squamous cell carcinoma (SCC) involving the labial commissure who underwent a A-EF, SFIF, PMMF, or TIMF for reconstructing through-and-through cheek defects involving the labial commissure following radical resection, between January 2012 and June 2017 at Sun Yat-sen Memorial Hospital, Sun Yat-sen University. The Institutional Review Board of Sun Yat-sen University approved this study. The patients included 20 men and 15 women ranging in age from 44 to 81 (median 59.1) years. According to the 2010 American Joint Committee on Cancer staging guidelines [13], the clinical stages of the disease or recurrence (rCS) were I, II, III + rCS III, and IV + rCS IV in 0, 8 (22.9%), 11 + 7 (51.4%), and 4 + 5 (25.7%) patients, respectively. The recurrences were classified as rCS III in 7 patients and rCS IV in 5. Eight patients were treated with surgery, including ipsilateral radical neck dissection (6 cases) and reconstruction with a pectoralis major myocutaneous flap (4 cases), forearm free flap (4 cases) or Abbe–Estlander flap (1 case), three patients underwent radiotherapy and chemotherapy and one patient underwent radiotherapy alone.

All cases of buccal SCC involving the labial commissure SCC underwent tumor resection and a partial maxillotomy plus marginal mandibulotomy was performed in 14 cases and a total maxillotomy plus total mandibulotomy in six cases; ipsilateral radical neck dissection was performed in 21 cases. The inner dimensions (in cm) of the A-EF, SFIF, PMMF and TIMF skin paddle were 1 × 2 to 2 × 3 (median 1.8 × 2.2), 5 × 3 to 7 × 5 (median 5.5 × 4.3), 6 × 4 to 7 × 5 (median 6.3 × 4.5), and 6 × 6 to 15 × 8 (median 9.8 × 6.7), respectively. The outer dimensions (in cm) were 2 × 2–3 × 5 (median 1.8 × 3.8), 5 × 4–7 × 8 (median 5.5 × 4.6), 6 × 6–7 × 6 (median 6.3 × 6.6), and 6 × 8–15 × 20 (median 9.8 × 13.2), respectively. The mean flap harvesting times in the A-EF, SFIF, PMMF and TIMF groups were 20, 55, 56 and 65 min, respectively. All patients were followed for at least 3 months postoperatively by a panel of three surgeons to assess the esthetic results, orbicularis oris function, and speech function. The esthetic result for the cheek and lip was rated as 1 = unsatisfactory, 2 = satisfactory, and 3 = excellent [14]. The orbicularis oris function was rated as 1 = unable to suction water with a straw, 2 = can suction some water with a straw, and 3 = can suction water with a straw [15]. The speech function was rated as 1 = slurred speech, 2 = intelligible speech, and 3 = normal speech. Table 1 summarizes the data for the A-EF, SFIF, PMMF, and TIMF groups.

**Table 1:** Demographics, clinical characteristics, and outcomes of the A-EF, SFIF, PMMF, and TIMF for reconstructing through-and-through cheek defects involving the labial commissure following cheek cancer ablation in 35 patients with buccal squamous cell carcinoma

	A-EF (*n* = 5) No. of patients (%)	SFIF (*n* = 8) No. of patients (%)	PMMF (*n* = 9) No. of patients (%)	TIMF (*n* = 13) No. of patients (%)	*P*-value
Sex					
Male	3 (80.0)	5 (62.6)	6 (66.7)	7 (53.8)	0.887^a^
Female	2 (20.0)	3 (37.4)	3 (33.3)	6 (46.2)	
Age, years (mean ± SD)	52.0 ± 8.5	64.3 ± 9.2	59.6 ± 8.2	59.8 ± 12.7	0.863^a^
Clinical stage					
I	0 (0.0)	0 (0.0)	0 (0.0)	0 (0.0)	0.026^b^
II	5 (100.0)	3 (22.5)	0 (0.0)	0 (0.0)	
III + rCS III	0 (0.0)	5 (42.5)	5 + 1 (66.7)	1 + 6 (53.8)	
IV + rCS IV	0 (0.0)	0 (0.0)	2 + 1 (33.3)	4 + 2 (46.2)	
Treatment					
TR	5 (100.0)	7 (87.5)	3 (33.3)	0 (0.0)	0.036^b^
TR + PM + MM	0 (0.0)	1 (12.5)	5 (55.6)	8 (61.5)	
TR + Man+Max	0 (0.0)	0 (0.0)	1 (11.1)	5 (38.5)	
Flap (cm)					
Inner dimensions					
Range, median	1 × 2 to 2 × 3, 1.8 × 2.2	5 × 3 to 7 × 5, 5.5 × 4.3	6 × 4 to 7 × 5, 6.3 × 4.5	6 × 6 to 15 × 8, 9.8 × 6.7	0.041^b^
Outer dimensions					
Range, median	2 × 2 to 3 × 5, 1.8 × 3.8	5 × 4 to 7 × 8, 5.5 × 4.6	6 × 6 to 7 × 6, 6.3 × 6.6	6 × 8 to 15 × 20, 9.8 × 13.2	0.033^b^
Successful (no.)	5 (100.0)	7 (87.5)	9(100.0)	13 (100.0)	0.967^a^
Local complications					
Hemorrhage	1 (20.0)	1 (12.5)	0 (0.0)	1 (7.7)	0.554^c^
Orocutaneous fistula	0 (0.0)	1 (12.5)	0 (0.0)	0 (0.0)	
Dehiscence in donor-site	0 (0.0)	0 (0.0)	1 (11.1)	2 (15.4)	
Esthetic results					
1	0 (0.0)	1 (12.5)	1 (11.1)	4 (30.8)	0.039^c^
2	1 (20.0)	2 (25.0)	6 (66.7)	7 (53.8)	
3	4 (80.0)	5 (62.5)	2 (22.2)	2 (15.4)	
Orbicularis oris function					
1	0 (0.0)	1 (12.5)	2 (22.2)	4 (30.8)	0.042^c^
2	0 (0.0)	1 (12.5)	4 (44.5)	6 (46.2)	
3	5 (100.0)	6 (75.0)	3 (33.3)	3 (23.0)	
Speech function					
1	0 (0.0)	0 (0.0)	2 (22.2)	5 (38.4)	0.046^c^
2	0 (0.0)	2 (25.0)	3 (33.3)	6 (46.2)	
3	5 (100.0)	6 (75.0)	4 (44.5.)	2 (15.4)	
Follow-up range, median (months)	6–38, 26.8	6–36, 25.0	6–32, 22.1	6–33, 20.8	0.829^a^
Status (months)					
AND	4 (80.0)	7 (87.5)	5 (55.6)	5 (38.4)	0.039^b^
AWD	1 (20.0)	1 (12.5)	2 (22.2)	4 (30.8)	
DOD	0 (0.0)	0 (0.0)	2 (22.2)	4 (30.8)	

Abbreviations: *A-EF* Abbe–Estlander flap, *SFIF* folded extended supraclavicular fasciocutaneous island flap, *PMMF* pectoralis major muscle flap, *TIMF* folded extended vertical lower trapezius island myocutaneous flap, *rCS* clinical staging of recurrence, *TR* tumor resection, *Man* mandibulotomy, *Max* maxillotomy, *PM + MM* partial maxillotomy plus marginal mandibulotomy, *AND* alive with no disease, *AWD* alive with disease, *DOD* died of disease

^a^ All groups were compared

^b^ The A-EF and SFIF groups were compared with the PMMF and TIMF groups

^c^ The A-EF group was compared with the SFIF, PMMF and TIMF groups

The statistical analyses were performed using SPSS 20 (IBM, Armonk, NY, USA). The chi-squared test, independent samples *t*-test, and Mann–Whitney *U*-test were used to analyze the data, and the level of significance was set at *P* < 0.*05*.

## Case reports

### Case 1

**Fig. 1:**
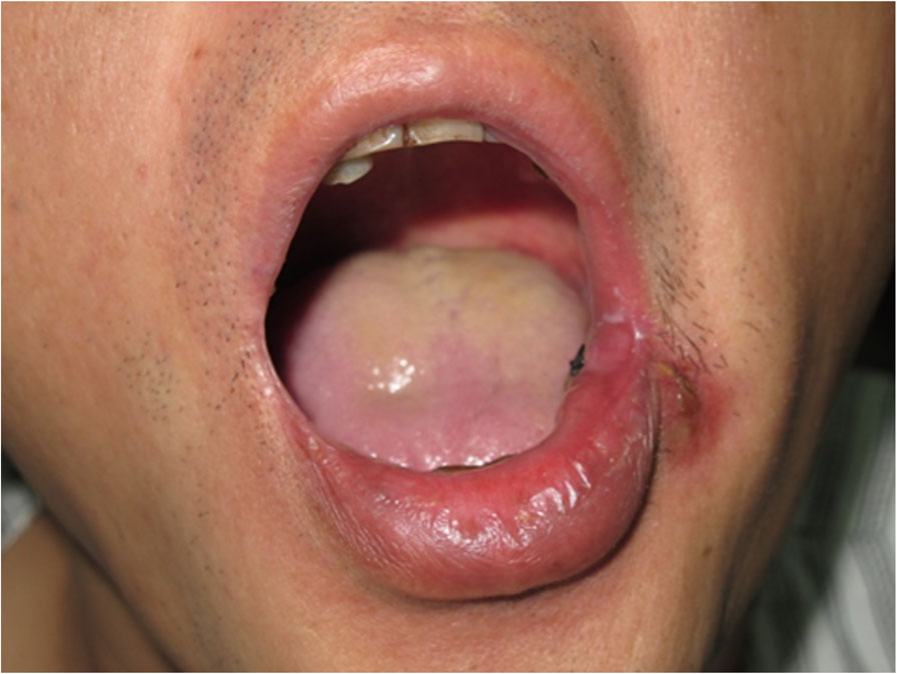
A 45-year-old man with stage II buccal squamous cell carcinoma involving the labial commissure

**Fig. 2:**
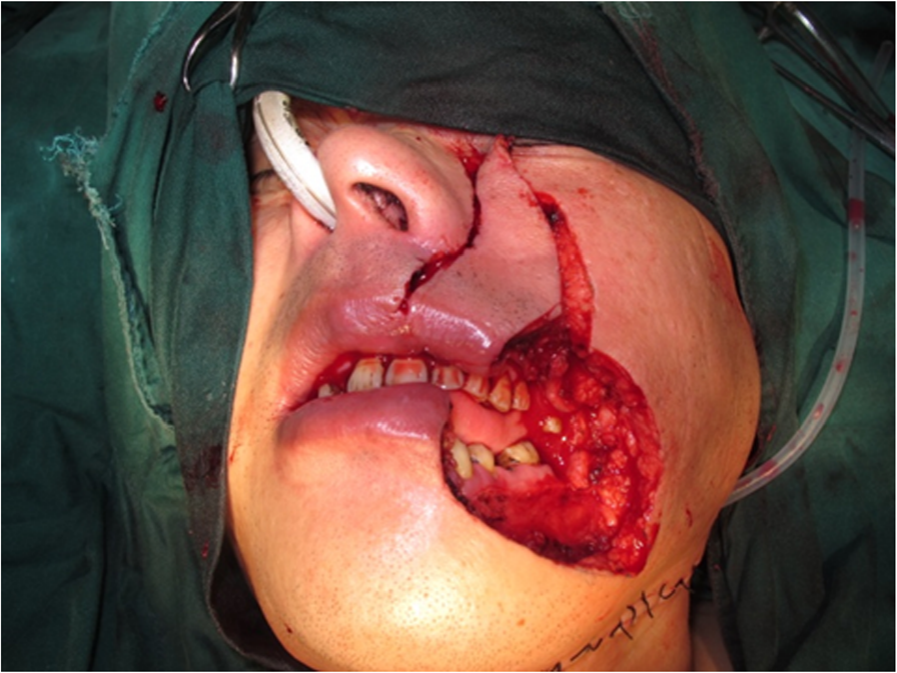
Defect in the cheek and adjacent oral commissure after tumor resection, with the Abbe–Estlander flap prepared along the nasolabial fold, creating a triangular total-thickness flap, based on the upper labial artery

**Fig. 3:**
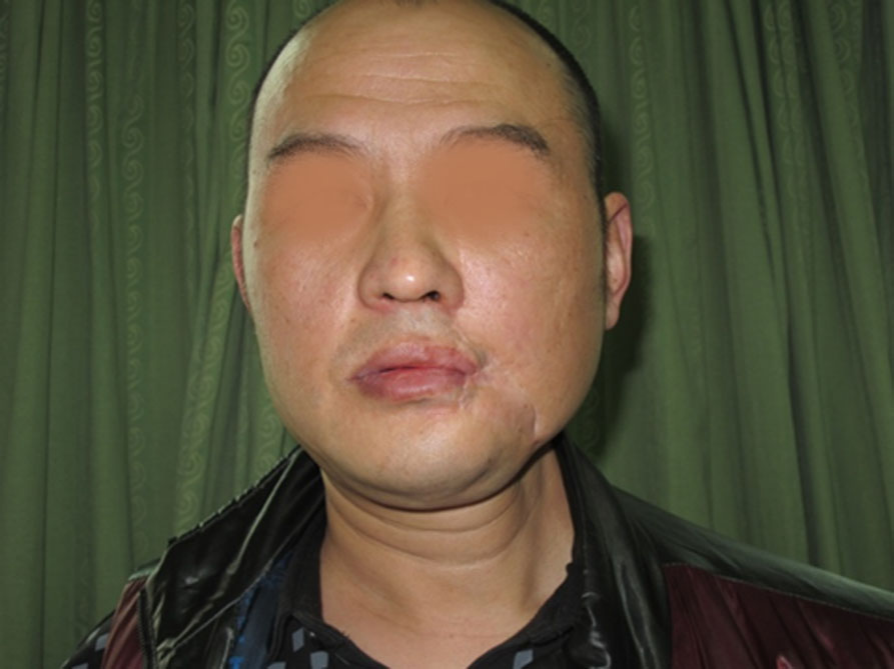
The postoperative appearance

A 45-year-old man presented with stage II buccal SCC involving the labial commissure (Fig. 1). Under general anesthesia, the A-EF was drawn along the nasolabial fold, creating a triangular total thickness flap; the flap was used to reconstruct the defect in the commissure following tumor resection and radical neck dissection (Fig. 2). The inner and outer dimensions of the A-EF skin paddle were 1.8 × 3.0 and 1.8 × 2.5 cm, respectively. At the 3-month follow-up, the esthetic result for the cheek and lip was excellent, the orbicularis oris function was rated 3 (can suction water with a straw), and the speech function was rated 3 (normal speech) (Fig. 3). At 23 months, the patient had local recurrence and underwent salvage surgery.

### Case 2

**Fig. 4:**
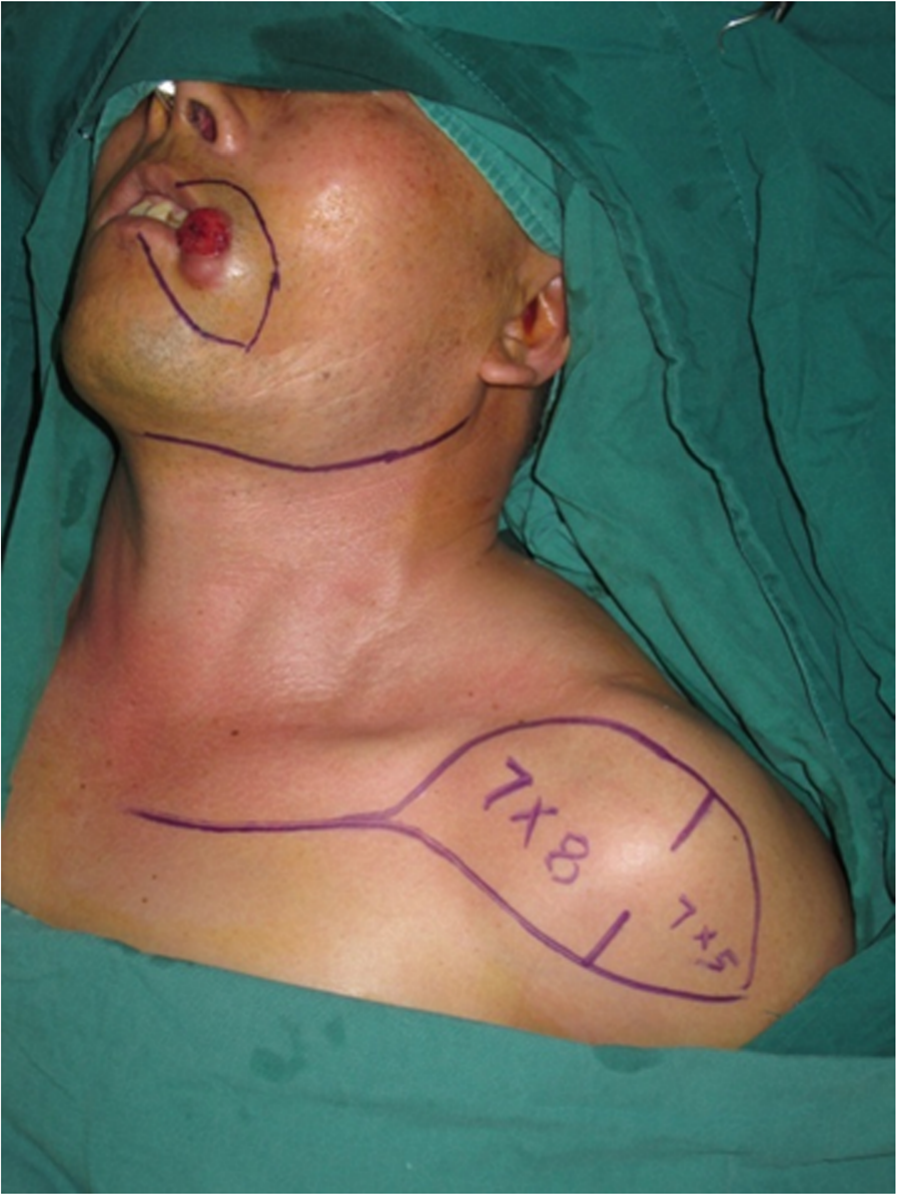
A 43-year-old man with stage III buccal squamous cell carcinoma involving the labial commissure. The incisions for the folded extended supraclavicular fasciocutaneous island flap (SFIF) and tumor excision have been drawn

**Fig. 5:**
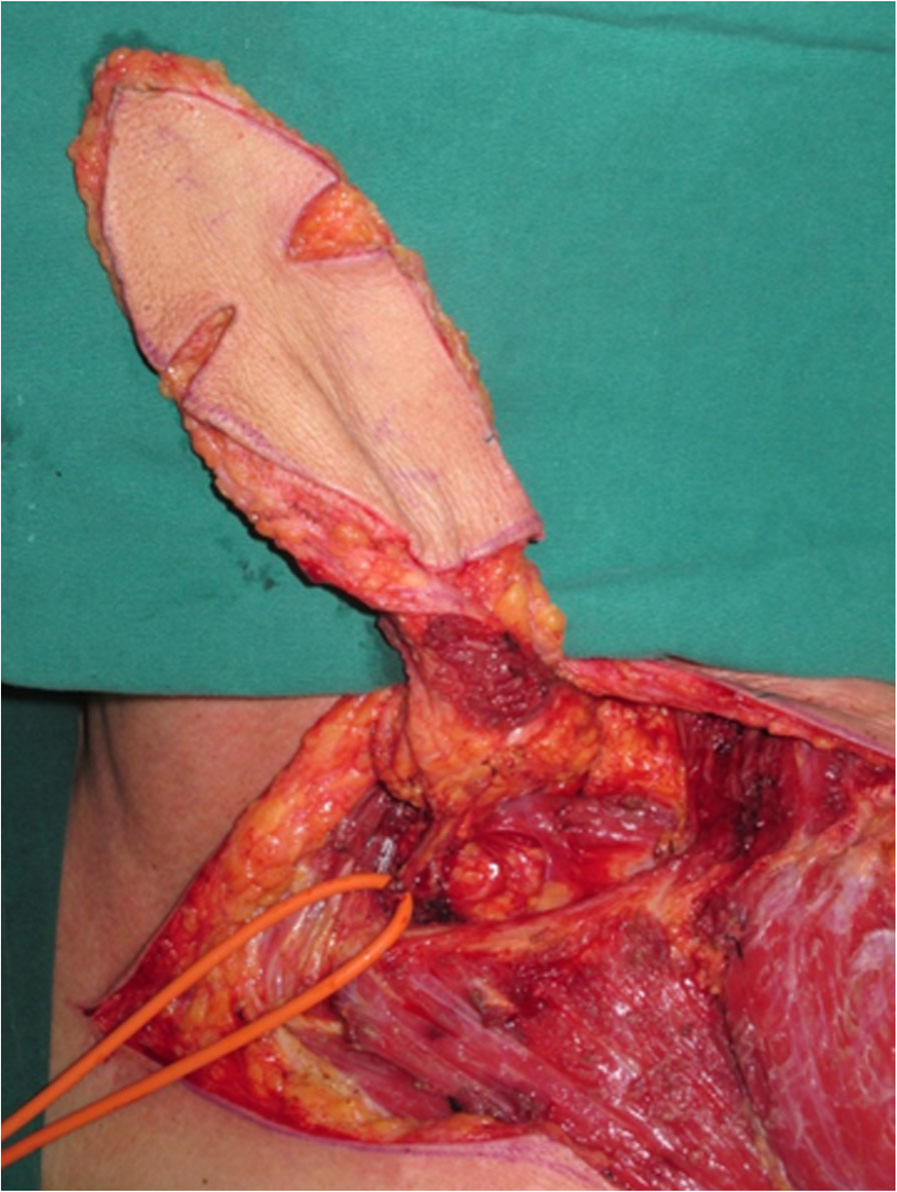
A foldable SFIF was created by dissecting the skin in the flap bilaterally

**Fig. 6:**
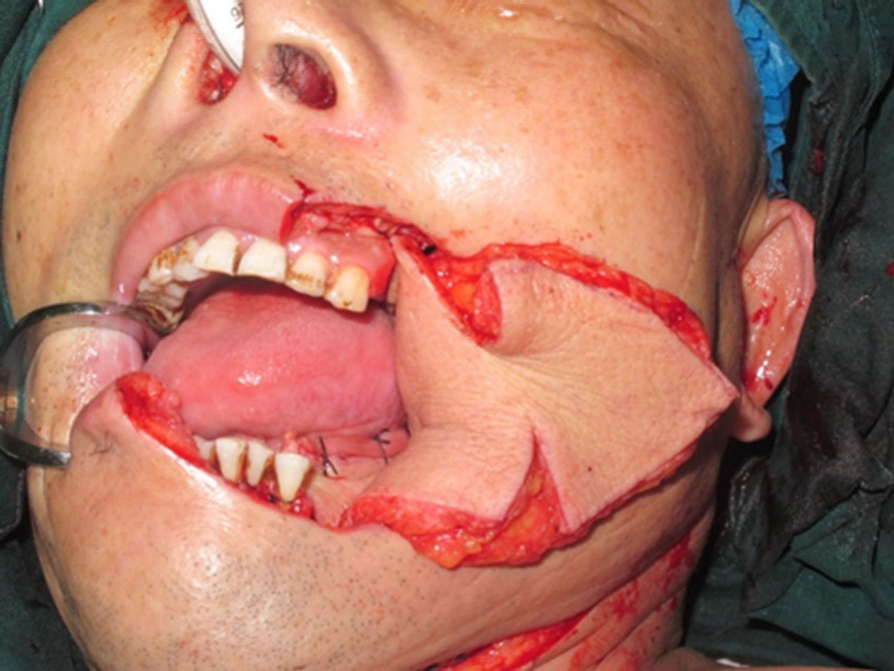
The medial portion of the flap was used for the buccal mucosa and the distal portion was used for the skin of the cheek

**Fig. 7:**
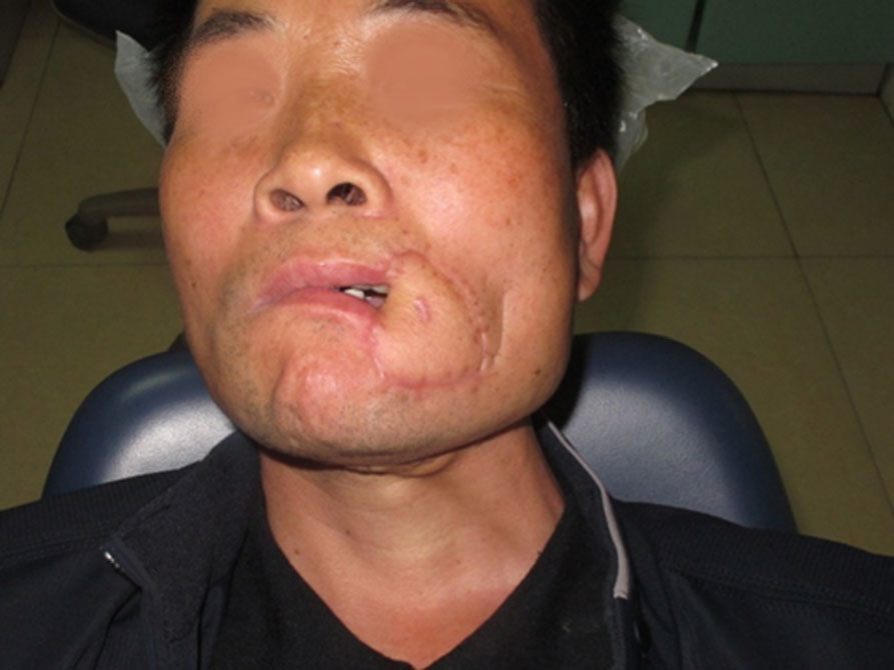
The postoperative appearance

A 43-year-old man presented with stage III buccal SCC involving the labial commissure. Under general anesthesia, he was placed on his side at an angle of approximately 45° with the head and neck extended moderately. Tumor resection and neck dissection were performed in this position. The folded extended SFIF based on the transverse cervical vessels and incisions for the tumor resection are shown in Fig. 4. A foldable flap with a skin paddle including inner (7 × 5 cm) and outer (7 × 8 cm) linings for reconstructing the full cheek defect and labial commissure were created by dissecting the skin in the flap bilaterally (Fig. 5). The flap was pulled through a tunnel to reach distant cheek defects; the medial portion of the flap was used for the buccal mucosa and the distal portion was used for the skin of the cheek following tumor resection and neck dissection (Fig. 6). The donor site was closed directly. At the 6-month follow-up, the esthetic result for the cheek and lip was satisfactory; the orbicularis oris function was rated 2 (can suction some water with a straw), and the speech function was rated 2 (intelligible speech) (Fig. 7). The patient was alive with no evidence of disease at 26 months.

### Case 3

**Fig. 8:**
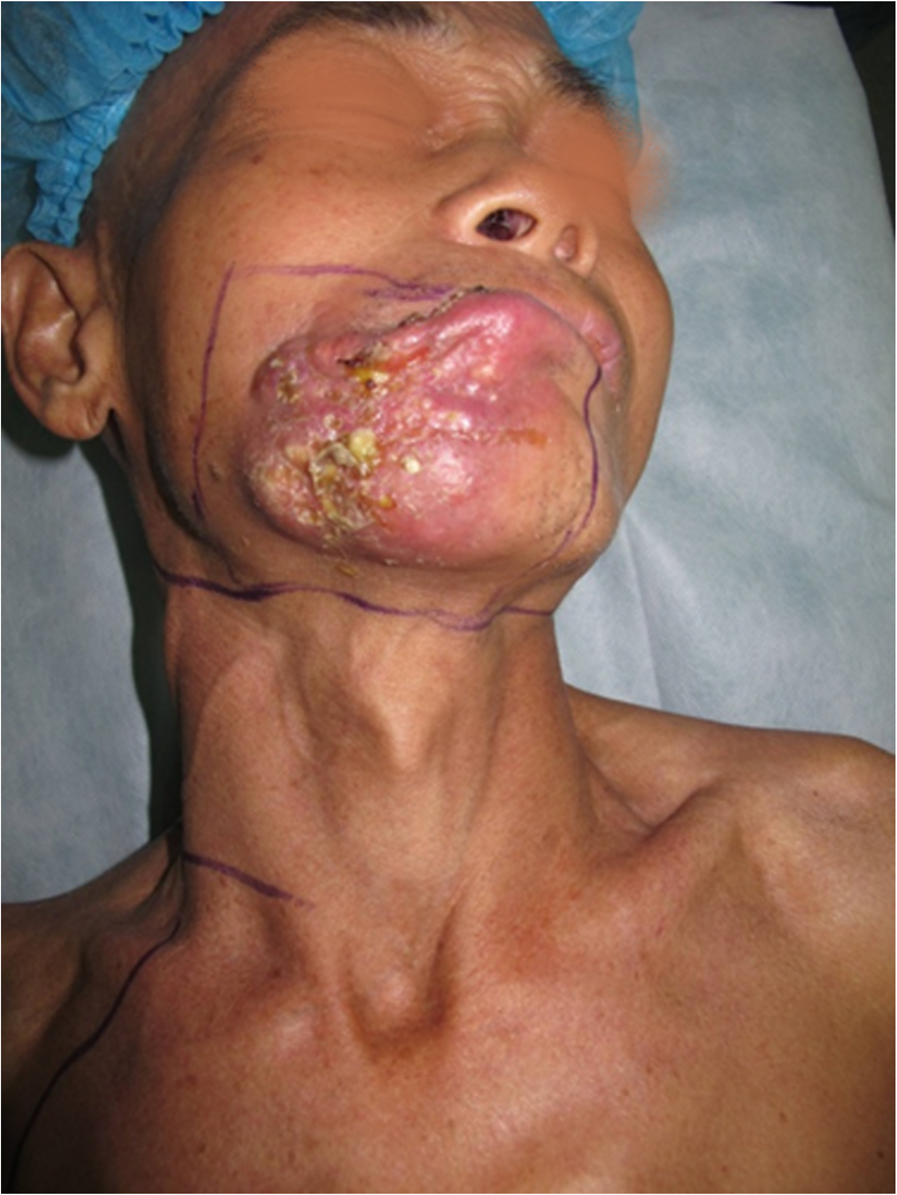
A 63-year-old man presented with stage IV buccal squamous cell carcinoma involving the labial commissure

**Fig. 9:**
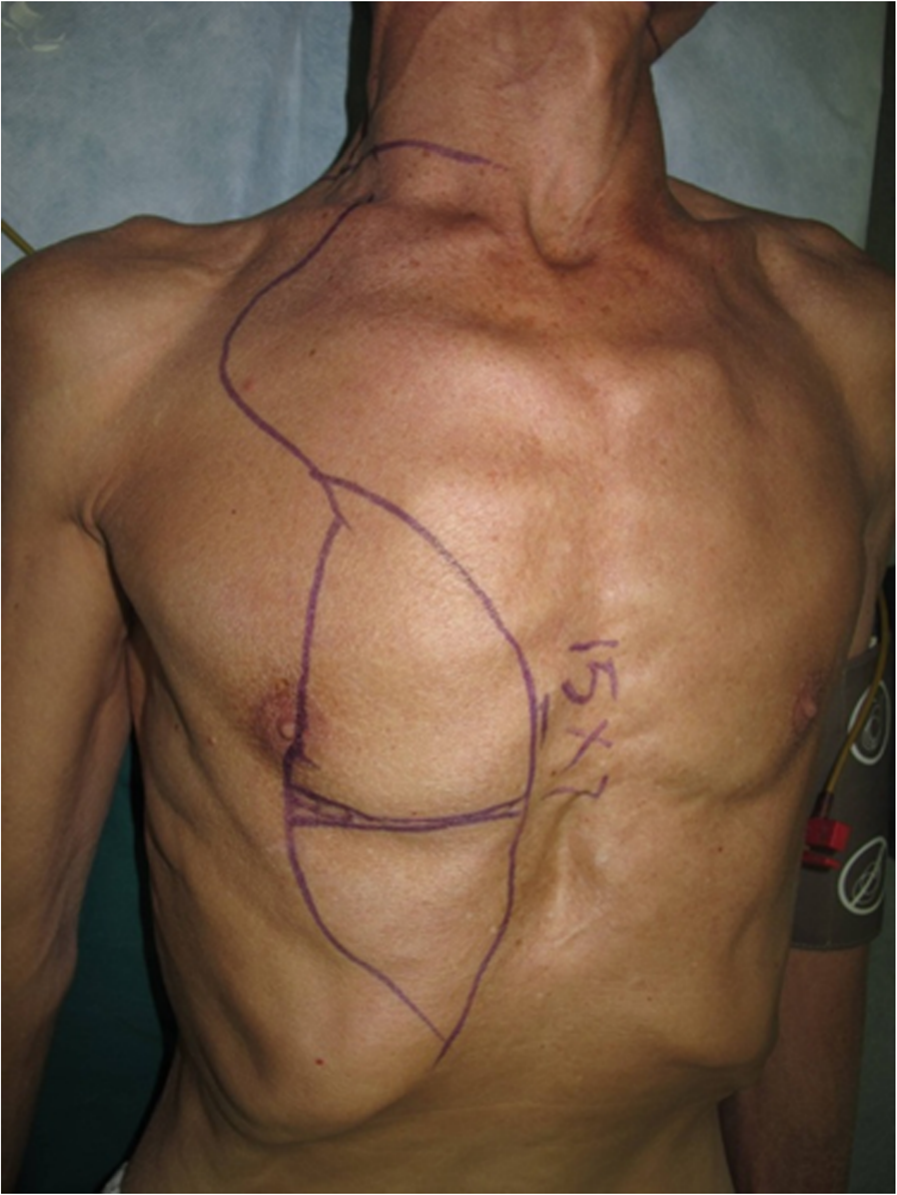
A folded pectoralis major muscle flap was designed

**Fig. 10:**
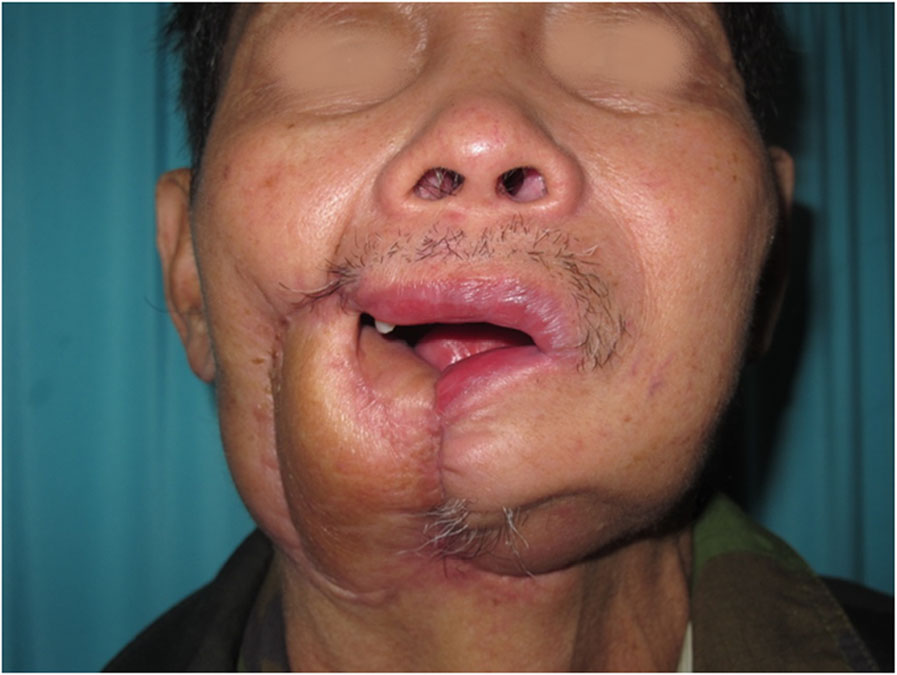
The postoperative appearance

A 63-year-old man presented with stage IV buccal SCC involving the labial commissure (Fig. 8). Under general anesthesia, a folded PMMF was used to reconstruct the defect in the commissure (Fig. 9). The flap was based on the thoracoacromial vessels and pulled through a tunnel to reach the distant cheek defect following tumor resection, with a partial maxillotomy plus marginal mandibulotomy and radical neck dissection. At the 4-month follow-up, the esthetic result for the cheek and lip was satisfactory; the orbicularis oris function was rated 2 (can suction some water with a straw) and the speech function was rated 2 (intelligible speech) (Fig. 10). The patient was alive with no evidence of disease at 22 months.

### Case 4

**Fig. 11:**
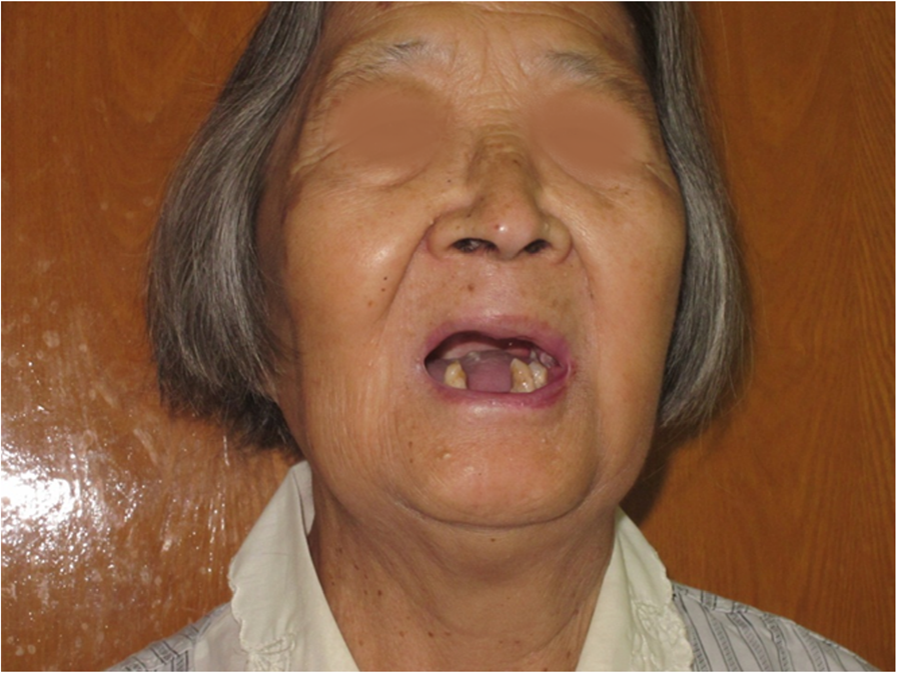
An 81-year-old woman with stage IV recurrent buccal squamous cell carcinoma involving the labial commissure

**Fig. 12:**
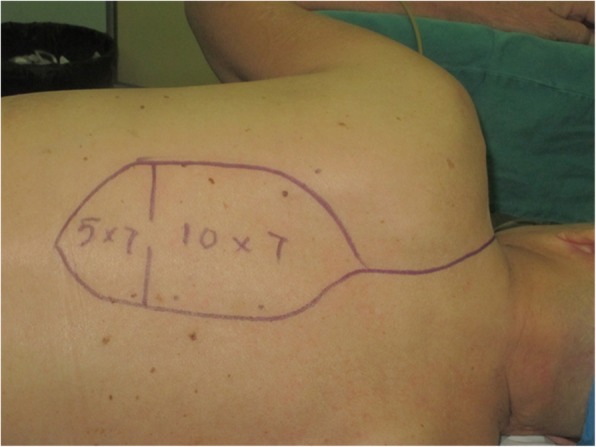
An extended vertical lower trapezius island myocutaneous flap (TIMF) with a skin paddle measuring (10 + 7) × 7 cm was designed

**Fig. 13:**
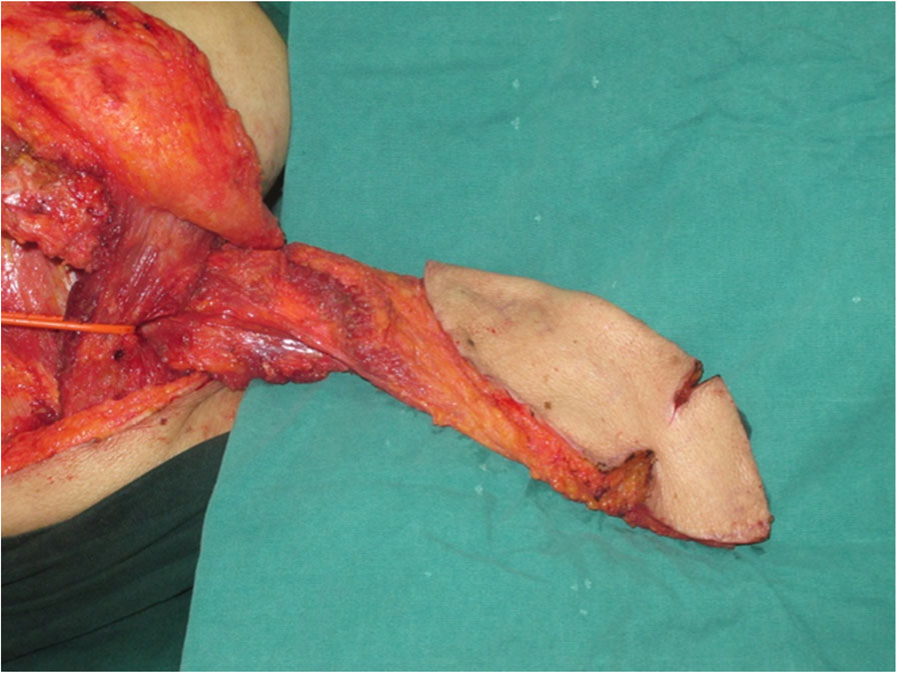
A foldable TIMF was created and the flap was passed through a tunnel in the upper part of the trapezius muscle

**Fig. 14:**
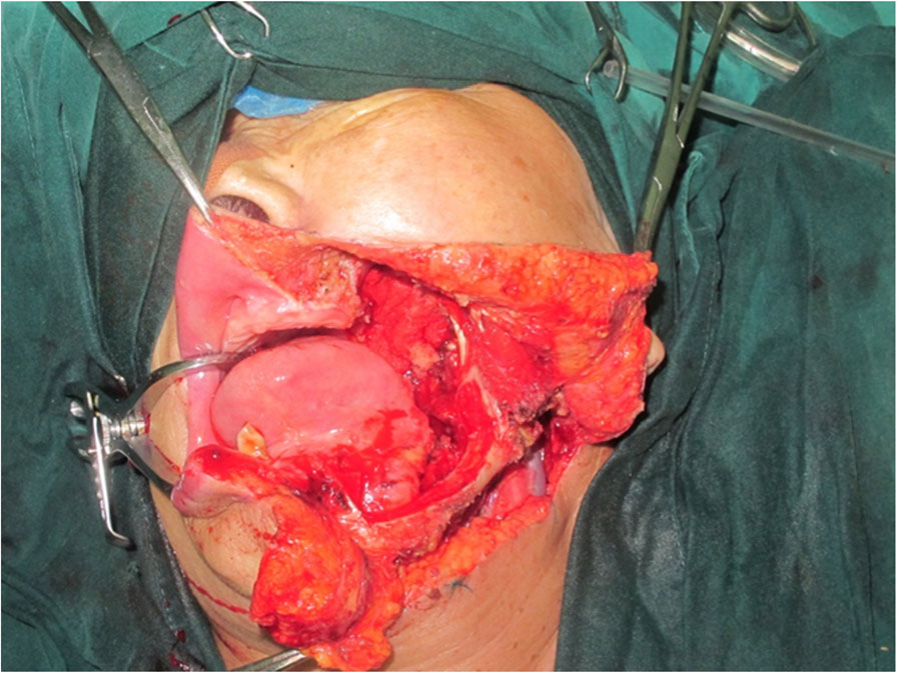
Wide excision of the tumor was made

**Fig. 15:**
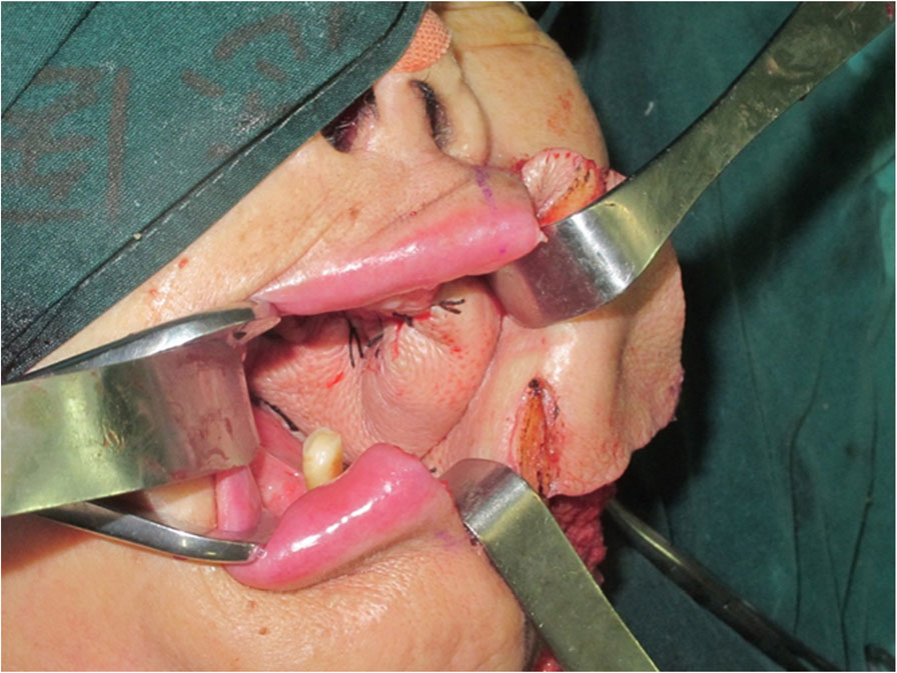
The distal portion of the flap was turned to serve as the mucosa of the mouth and the medial portion for the skin

**Fig. 16:**
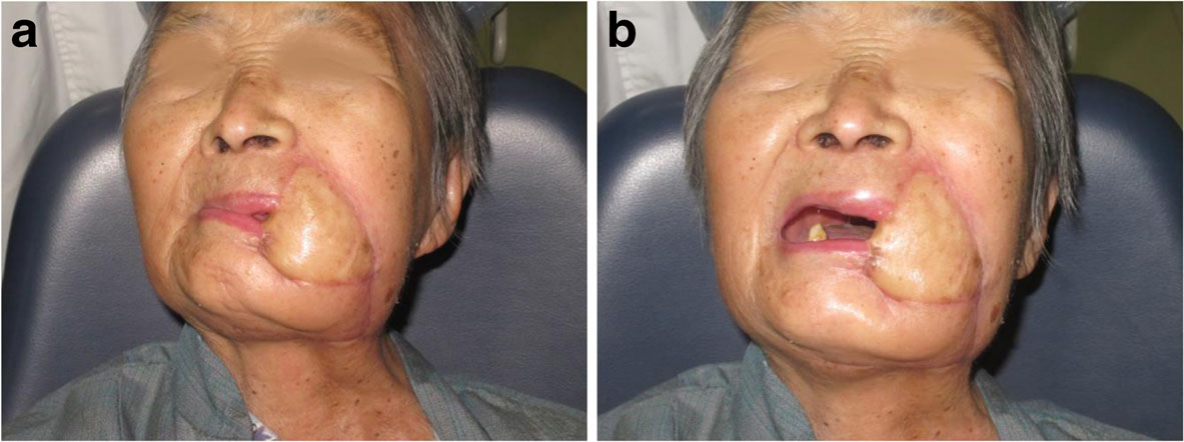
The view 3 months after surgery: (**a**) closed and (**b**) open mouth

An 81-year-old woman presented with stage IV recurrent buccal SCC involving the labial commissure (Fig. 11). Under general anesthesia, a folded extended vertical lower TIMF based on the transverse cervical vessels was raised with the patient in the lateral prone position. The flap was designed to follow the course of the transverse cervical vessels, ensuring that the center of the long axis of the flap was between the vertebral column and the medial border of the scapula (Fig. 12). The flap was raised from its midpoint and proceeded in a medial to lateral direction, preserving the upper part of the trapezius muscle; a tunnel was made in the upper part of the trapezius muscle. A foldable flap with a skin paddle including inner (7 × 5 cm) and outer (7 × 10 cm) linings for reconstructing the full cheek defect and labial commissure was created by dissecting the skin in the flap bilaterally (Fig. 13). The donor area was closed primarily. After tumor resection, a partial maxillotomy plus marginal mandibulotomy and radical neck dissection were performed (Fig. 14). The distal portion of the flap was turned to serve as the inner lining or oral mucosa, and the medial portion for the outer lining or skin (Fig. 15). At the 3-month follow-up, the esthetic result for the cheek and lip was satisfactory; the orbicularis oris function was rated 2 (can suction some water with a straw), and the speech function was rated 2 (intelligible speech) (Fig. 16). The patient was alive with no evidence of disease at 27 months.

## Results

The study included 35 patients (21 men and 14 women). There were no significant differences among the A-EF, SFIF, PMMF, and TIMF groups in terms of age and sex. However, the A-EF and SFIF groups differed significantly (*P* < 0.05) from the PMMF and TIMF groups in terms of tumor clinical stage and type of treatment. The inner dimensions of the PMMF (median 6.3 × 4.5) and TIMF (median 9.8 × 6.7) skin paddles were larger than those of the A-EF (median 1.8 × 2.2) and SFIF (median 5.5 × 4.3) groups (*P* < 0.05). The outer dimensions of the PMMF (median 6.3 × 6.6) and TIMF (median 9.8 × 13.2) were also larger than those of the A-EF (median 1.8 × 3.8) and SFIF (median 5.5 × 4.6) groups (*P* < 0.05). The mean flap harvesting times in the SFIF, PMMF, and TIMF groups were longer than in the A-EF groups. No major complications developed in any patient. One flap failures occurred and no significant difference was observed in the rate of flap among the A-EF, SFIF, PMMF, and TIMF groups. Hemorrhage, orocutaneous fistulas and dehiscence in donor-site occurred in one (20%), two (21.5%), one (11.1%), and three (23.1%) cases in the A-EF, SFIF, PMMF, and TIMF groups, respectively. An urgent exploratory operation was performed to stop the bleeding when a hemorrhage occurred. The flap failures, orocutaneous fistulas and dehiscence in donor-site were treated successfully with debridement. The esthetic results, orbicularis oris function, and speech function were significantly (*P* < 0.05) better in the A-EF group than in the SFIF, PMMF, and TIMF groups. The patients were followed for 6–38 months (median 26.8, 25.0, 22.1, and 20.8 months in the A-EF, SFIF, PMMF, and TIMF groups, respectively). At the final follow-up, 4 (80.0%) patients in the A-EF, 7 (87.5%) in the SFIF, 5 (55.6%) in the PMMF, and 5(38.4%) in the TIMF groups were alive with no disease, while 1 (20.0%), 1 (22.2%), 2 (22.2%), and 4 (30.8%), respectively, were alive with disease. Two (22.2%) patients in the PMMF and 4 (30.8%) in the TIMF group had died of local recurrence or distant metastases at between 9 and 38 months. There was a significant (*P* < 0.05) survival difference in the A-EF and SFIF groups compared with the PMMF and TIMF groups. Table 1 summarizes the data for the four groups.

## Discussion

Pedicle flaps play an important role in head-and-neck reconstruction, even in the era of microvascular surgery [9–12, 16, 17], not only as an alternative to a free flap but also because they involve less risk to the patient. In this study, we compared the outcomes of four types of pedicle flap for reconstructing through-and-through cheek defects involving the labial commissure following cheek cancer ablation: the A-EF based on the superior labial vessels; the SFIF based on the transverse cervical vessels; the PMMF based on the thoracoacromial vessels; and the TIMF based on the transverse cervical vessels. The four groups were similar in terms of patient age and sex, and the success rates of the four types of pedicle flap were very high and there were no significant differences in the rate of flap failure among the four groups. The four types of pedicle flap were reliable and safe. Our results showed that the patients in the PMMF and TIMF groups were at more advanced tumor stages compared with the A-EF and SFIF groups, the dissections were wider. Consequently, the dimensions of the inner and outer linings of the PMMF and TIMF skin paddles were larger than for the A-EF and SFIF groups. We believe that four types of flap have major roles in surgery for different clinical stages: the A-EF is suitable for reconstructing defects in clinical stage II disease; the SFIF for clinical stage II or III disease; the PMMF for clinical stage III or IV disease; and the TIMF for stage rCS III or rCS IV disease. The esthetic results, orbicularis oris function, and speech function were significantly better in the A-EF group than in the other three groups, which might be related to the less advanced clinical stage. As the patients in the PMMF and TIMF groups had more advanced tumors, there was a significant survival difference in the A-EF and SFIF groups compared with the PMMF and TIMF groups. We believe that when an extended vertical lower TIMF is used to reconstruct the major defects following wide resection of a tumor or craniofacial resection, the resulting swallowing function, speech function, and appearance of the face and neck were acceptable. Salvage surgery remains an effective treatment modality for selected patients with advanced recurrent oral and oropharyngeal SCC, and the extended vertical lower TIMF is a large, simple, and reliable flap for reconstructing the major defect following salvage surgery [10, 12, 18].

## Conclusions

The A-EF is suitable for reconstructing defects of clinical stage II disease; the SFIF for clinical stage II or III disease; the PMMF for clinical stage III or IV; and the TIMF for clinical stage rCS III or rCS IV disease.
